# Supplementary material for: COZOID: contact zone identifier for visual analysis of protein-protein interactions
Source: BMC Bioinformatics. 2018 Apr 6;19:125. doi: 10.1186/s12859-018-2113-6 (PMC5889581; doi:10.1186/s12859-018-2113-6)
Supplement: Supplementary file 2 — Software build. Executable binary file of the software tool. (ZIP 118,784 kb) [file 12859_2018_2113_MOESM2_ESM.zip › cozoid/java/docs/beansbinding-1.2.1-doc.zip!/org/jdesktop/swingbinding/adapters/JTextComponentAdapterProvider.html]

JTextComponentAdapterProvider (Beans Binding)


---


|  |  |  |
| --- | --- | --- |
| **Overview** \| **Package** \| **Class** \| **Use** \| **Tree** \| **Deprecated** \| **Index** \| **Help** | |  |
| **PREV CLASS**   **NEXT CLASS** | **FRAMES**    **NO FRAMES**     **All Classes** |  |
| SUMMARY: NESTED \| FIELD \| CONSTR \| METHOD | DETAIL: FIELD \| CONSTR \| METHOD |  |


---


## org.jdesktop.swingbinding.adapters Class JTextComponentAdapterProvider

```
java.lang.Object
  org.jdesktop.swingbinding.adapters.JTextComponentAdapterProvider
```

**All Implemented Interfaces:**: BeanAdapterProvider

---

``` public final class JTextComponentAdapterProvider extends java.lang.Object implements BeanAdapterProvider ```

---

| **Nested Class Summary** | |
| --- | --- |
| `class` | `JTextComponentAdapterProvider.Adapter` |


**Constructor Summary**

`JTextComponentAdapterProvider()`


| **Method Summary** | |
| --- | --- |
| `java.lang.Object` | `createAdapter(java.lang.Object source, java.lang.String property)` |
| `java.lang.Class<?>` | `getAdapterClass(java.lang.Class<?> type)` |
| `boolean` | `providesAdapter(java.lang.Class<?> type, java.lang.String property)` |

**Methods inherited from class java.lang.Object**

`clone, equals, finalize, getClass, hashCode, notify, notifyAll, toString, wait, wait, wait`

**Constructor Detail**

### JTextComponentAdapterProvider

```
public JTextComponentAdapterProvider()
```


**Method Detail**

### providesAdapter

```
public boolean providesAdapter(java.lang.Class<?> type,
                               java.lang.String property)
```

:   **Specified by:**: `providesAdapter` in interface `BeanAdapterProvider`

---


### createAdapter

```
public java.lang.Object createAdapter(java.lang.Object source,
                                      java.lang.String property)
```

:   **Specified by:**: `createAdapter` in interface `BeanAdapterProvider`

---


### getAdapterClass

```
public java.lang.Class<?> getAdapterClass(java.lang.Class<?> type)
```

:   **Specified by:**: `getAdapterClass` in interface `BeanAdapterProvider`


---


|  |  |  |
| --- | --- | --- |
| **Overview** \| **Package** \| **Class** \| **Use** \| **Tree** \| **Deprecated** \| **Index** \| **Help** | |  |
| **PREV CLASS**   **NEXT CLASS** | **FRAMES**    **NO FRAMES**     **All Classes** |  |
| SUMMARY: NESTED \| FIELD \| CONSTR \| METHOD | DETAIL: FIELD \| CONSTR \| METHOD |  |


---
